# Nanomaterial-based sensors for microbe detection: a review

**DOI:** 10.1186/s11671-024-04065-x

**Published:** 2024-07-30

**Authors:** Muhammad Qamar Khan, Jahangir Khan, Muhammad Abbas Haider Alvi, Hifza Nawaz, Muhammad Fahad, Muhammad Umar

**Affiliations:** 1 Department of Textile Engineering, School of Engineering and Technology, National Textile University, Faisalabad, 37610 Punjab Pakistan; 2 Department of Materials, The University of Manchester, Manchester, M13 9PL UK

**Keywords:** Microbes sensor, Nanofibers, Colorimetric phenomenon, Biosensor

## Abstract

Airborne microorganisms pose a significant health threat, causing various illnesses. Traditional detection methods are often slow and complex. This review highlights the potential of nanomaterial-based biosensors, particularly colorimetric sensors, for rapid and on-site detection of airborne microbes. Colorimetric sensors offer real-time visual detection without complex instrumentation. We explore the integration of these sensors with Lab-on-a-Chip technology using PDMS microfluidics. This review also proposes a novel PDMS-based colorimetric biosensor for real-time detection of airborne microbes. The sensor utilizes a color change phenomenon easily observable with the naked eye, simplifying analysis and potentially enabling point-of-care applications.

**Graphical Abstract:**

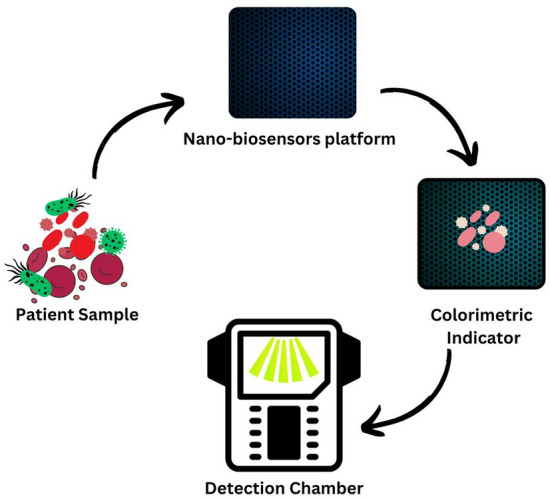

## Introduction

Nanomaterials are proving that they can help in the identification of bacteria and viruses in the ongoing battles against infectious diseases. The distinctive physical and electronic properties in comparison to the bulk counterparts are beneficial for biosensing. Another area of use is in colorimetric based detection where an agent changes color in the presence of a pathogen. Gold nanoparticles are staring, and the metal nanoparticles are again the most important here. It has been discovered that their surfaces can be painted with antibodies or particularly DNA sequences that only fix onto a target microbe or virus. In this case, the nanoparticles agglomerate and become detain, which changes the color observed visually. Unlike other similar technologies, its plain to read display requires very little complex equipment which is highly beneficial for field uses. Another interesting field can be associated with such entities as nanozymes, for instance, graphene oxide or iron oxide magnetic nanoparticles. These nanomaterials are used to imitate peroxidase enzyme, an enzyme that produces a colored product upon the reaction of a target. There is also the possibility to implement purely label-free detection employing nanozymes in this way. There is a potential in the use of nanomaterials for the identification of microbes and viruses. Such technologies can be useful in quickly detecting structural changes, high sensitivity, and small sizes of diagnostic equipment. This opens opportunities to perform the test in the nearest clinic or, in some cases, right at home, and detect the disease at an earlier stage, which is beneficial to the general health of people.

Fibers having a breadth under 100 nm are known as nanofibers [1]. There are several methods to fabricate nanofibers, but the most efficient and simple method is electrospinning which utilizes electrostatic forces to produce electro-spun nanofibers [2]. This technique accomplished significantly more consideration recently not just because of its flexibility in spinning a huge assortment of polymeric strands but additionally because of its ability to create persistent filaments in the nano range, which is generally hard to acquire strands by utilizing strands mechanical fiber turning innovations strategies [3]. Overall, this is a simple strategy to create nanofibers from a huge scope of polymers [4]. A simple electrospinning setup in the laboratory demands a leading gatherer, a level tip needle, a syringe, and a high-level voltage powerhouse [5].

Electrospun nanofibers have various characteristics including a tunable body, higher surface-to-volume proportion Geometric surface, permeability, flexibility to confirm the vast area of sizes and shapes, and to supervise the anatomy of nanofibers to achieve the required outcomes from its characteristics as well as physical, synthetic, and biotic properties increase their use in various fields [6]. Electrospun nanofibers have attractive applications these applications these nanofibers are used in several fields such as pharmaceutical composition [7], tissue engineering scaffolds [8], optical electronics [9], biotechnology [10], defense and security [11], environmental engineering [12], wound dressing [13], filter formation [14], energy storage [15], drug delivery [16], biomedical and health care applications [17] as well as in sensing [18].

Nowadays, nanofibers [19–23] are the center of attention for sensing properties. Sensing is converting a quantity that a person wants to measure into a usable signal (usually electronics). Nanosensors are used for monitoring chemical and physical phenomena in areas where hard to go [24]. Nanosensors can be classified based on (1) structure, (2) energy (3) and applications.

- Analysis based on structure: There are four kinds of sensors divided on the base of structure; (i) electromagnetic sensors (ii) optical sensors (iii) mechanical sensors/vibrational nanosensors (iv) chemical nanosensors.
- Classification as indicated by energy source: In this type, nanosensors are classified into two categories (i) passive nanosensors which require no energy source like piezoelectric sensors and thermocouples (ii) active nanosensors which require energy source like thermistors [25].
- Classification based on applications: There are four kinds of sensor categories based on applications (i) biosensors (ii) deployable nanosensors (iii) chemical sensors (iv) electrometers [26].

But in the present time, as the microbes are increasing day by day, there is need of reliable, efficient, and accurate detection systems to identify them in advance.. Nanomaterials are showing significant advantages in the design of novel biosensing systems. Nanobiosensors are highly active and low-cost devices for this purpose [27]. A nano-biosensor is a type of biosensor that incorporates nanomaterials in its design for detecting specific biological or chemical substances. These sensors combine a biological recognition element (such as enzymes, antibodies, or nucleic acids) with a transducer (such as electrical, optical, or mechanical) that translates the interaction between the target substance and the recognition element into a measurable signal. Nanomaterials used in these biosensors, such as nanoparticles, nanowires, or graphene, allow for enhanced sensitivity, specificity, and speed in detection due to their large surface area-to-volume ratio and unique properties [28]. The main role of nano biosensors is to sense a biological-specific material and in environmental monitoring. Often these materials are proteins, antibodies, enzymes, and bio molecules [29]. In environmental monitoring, these nano biosensors are utilized for the direct discovery of pollutants [30], Microbes detection [31] and so on. There are three main components of nano biosensors namely as bioreceptors, transducers, and the detector [32]. Many researchers worked on nanobiosensors which are shown in Table 1.

**Table 1:** Nanomaterials-based sensors

Nanomaterial	Detection Techniques	Target	Detection time (min)	Limit of Detection (LOD) and Sensitivity	Limitations	References
Au-CNT nanohybrid	Colorimetric	H3N2 influenza virus	10	LOD: 3.4 PFU/ml Sensitivity: 10–50,000 PFU/ml	Potential interference from other substances in complex samples	[33]
Graphene with gold nanoparticles	Colorimetric	Norovirus	5	LOD: 92.7 pg/mL Sensitivity: 100 pg/mL to 10 μg/mL	Tability of nanoparticles could be affected by environmental conditions	[34]
Graphene oxide with gold nanoparticles	Colorimetric analysis	RSV	20	LOD: 0.1 ng/mL < br > Sensitivity: 0.1 ng/mL to 1 μg/mL	Limited sensitivity and specificity	[35]
Gold nanoparticles	Colorimetric analysis	Avian H9N2 influenza virus	90	LOD: 1.0 ng/mL < br > Sensitivity: 1 ng/mL to 10 μg/mL	Time-consuming compared to other methods	[36]
AuCNT/fluoro-immunoassay AgNPs/ resonance energy transfer	Luminescence	influenza H3N2 virus influenza H1N1 Virus	60	LOD: 5 PFU/ml < br > Sensitivity: 5–10^6 PFU/ml	Requires specialized equipment and expertise	[37]
AgNPs	Surface enhanced-Raman spectroscopy	RSV	10	LOD: 0.2 PFU/mL < br > Sensitivity: 0.2–10^5 PFU/mL	High cost of silver nanoparticles and potential toxicity	[38]
CdSe-ZnS-QDs	SPR analysis	Norovirus	-	LOD: 10 ng/mL < br > Sensitivity: 10 ng/mL to 100 μg/mL	Complexity in preparation and potential toxicity	[39]
CdSe-ZnS-QDs	Fluorescence quenching analysis	Avian H5N1 influenza virus	30	LOD: 1.5 ng/mL < br > Sensitivity: 1.5 ng/mL to 15 μg/mL	Fluorescence quenching can be affected by environmental factors	[40]
AuNPs-CdSe/TeS-QDs alloyed	SPR analysis	Influenza H1N1 and Influenza H3N2 virus	15–20	LOD: 3 PFU/ml < br > Sensitivity: 3–10^6 PFU/ml	Potential for high background signal and difficulty in multiplexing	[41]
RNA conjugated AuNPs	Colorimetric	Influenza Virus	–	LOD: 10 PFU/ml < br > Sensitivity: 10–10^6 PFU/ml	Stability of RNA conjugates and potential degradation	[42]
Silicon dioxide with silver nanoparticles	MEF	Influenza H5N1 virus	30	LOD: 1.2 ng/mL < br > Sensitivity: 1.2 ng/mL to 12 μg/mL	Limited reusability and potential interference from biological samples	[43]
Nanowires of silicon	FET	Influenza H3N2 virus	1	LOD: 0.1 PFU/ml < br > Sensitivity: 0.1–10^5 PFU/ml	High fabrication cost and potential for signal drift over time	[44]
Nanowires of silicon	FET	Avian H1N1 influenza virus	30	LOD: 0.5 PFU/ml < br > Sensitivity: 0.5–10^6 PFU/ml	Susceptibility to environmental variations	[45]
A thin film of indium tin oxide	FET	Avian H5N1 influenza virus	-	LOD: 1 PFU/ml < br > Sensitivity: 1–10^6 PFU/ml	Fragility and potential for interference from other substances	[46]
Single-walled carbon nanotubes	FET	Avian H5N1 influenza virus	–	LOD: 2 PFU/ml < br > Sensitivity: 2–10^5 PFU/ml	Difficulty in mass production and uniformity of nanotubes	[47]
Nanotubes of carbon	FET	Influenza A virus	1	LOD: 0.05 PFU/ml < br > Sensitivity: 0.05–10^4 PFU/ml	High cost of production and difficulty in large-scale integration	[48]
Rolled-up nanomembrane electrodes	EIS analysis	Avian H1N1 Influenza virus	-	LOD: 1 ng/mL < br > Sensitivity: 1 ng/mL to 10 μg/mL	Potential for signal instability and difficulty in handling	[49]
Graphene Oxide	EIS analysis	Influenza H1N1 virus H1N1	> 47	LOD: 1 ng/mL < br > Sensitivity: 1 ng/mL to 100 μg/mL	Long detection time and potential for high background noise	[50]
Multiwalled carbon nanotubes with cobalt phthalocyanine and PAMAM nanocomposites on carbon electrode	EIS analysis	Avian H5N1 influenza virus	–	LOD: 2 ng/mL < br > Sensitivity: 2 ng/mL to 20 μg/mL	Complexity in preparation and potential for batch-to-batch variation	[51] [52]
Gold nanoparticles with bi-functional fluorescence magnetic nanospheres	LSV analysis	Avian H7N9 influenza virus	–	LOD: 0.1 ng/mL < br > Sensitivity: 0.1 ng/mL to 1 μg/mL	Potential for interference from other magnetic materials	[53]
rGO	amperometry	Influenza H1N1 virus	–	LOD: 2 ng/mL < br > Sensitivity: 2 ng/mL to 20 μg/mL	Potential for variability in graphene oxide properties	[54]
Carbon nanotubes with gold magnetic nanoparticles	LSV analysis	Norovirus and Influenza H1N1 virus	–	LOD: 0.5 ng/mL < br > Sensitivity: 0.5 ng/mL to 5 μg/mL	Complexity in synthesis and potential for signal interference	[55]
Graphene-based silver nanoparticles	LSV analysis	Avian influenza H7 virus	30	LOD: 1 ng/mL < br > Sensitivity: 1 ng/mL to 10 μg/mL	Stability issues and potential for silver toxicity	[56]
Nanoparticles of gold	DPV analysis	Avian H5N1 influenza virus	–	LOD: 0.2 ng/mL < br > Sensitivity: 0.2 ng/mL to 2 μg/mL	High cost and potential for non-specific binding	[57]
Nanostructures of graphene oxide	DPV analysis	Influenza H1N1 virus, Hemagglutinin H5N1 protein	< 1	LOD: 0.01 ng/mL < br > Sensitivity: 0.01 ng/mL to 1 μg/mL	Potential for non-specific adsorption and complex preparation	[58]
SWCNT	Differential pulse voltammetry	Influenza H1N1 virus	30	LOD: 0.5 ng/mL < br > Sensitivity: 0.5 ng/mL to 5 μg/mL	High cost and difficulty in handling single-walled carbon nanotubes	[59]
Gold nanoparticles with multi-wall carbon nanotubes and polypyrrole nanowires	DPV analysis	Avian influenza H5N1 virus	–	LOD: 1 ng/mL < br > Sensitivity: 1 ng/mL to 10 μg/mL	Complexity in preparation and potential for non-specific interactions	[60]
Multi-walled carbon nanotubes	Conductometric sensing analysis	Influenza type A virus	4	LOD: 0.2 ng/mL < br > Sensitivity: 0.2 ng/mL to 2 μg/mL	Potential for signal drift and difficulty in achieving high specificity	[61]
Nanotubes of carbon	Chemiresistive sensing analysis	Influenza H5N1 virus	15	LOD: 0.5 ng/mL < br > Sensitivity: 0.5 ng/mL to 5 μg/mL	Sensitivity to environmental factors and potential for non-specific binding	[62]
CORONAVIRUSES SARS- CoV						
SWCNTs	FET	Nucleocapsid N protein of the SARS virus	–	LOD: 0.1 ng/mL < br > Sensitivity: 0.1 ng/mL to 10 μg/mL	High cost and potential for non-specific binding	[63, 64]
Nanoparticles of gold	SPCEs	Sequence of SARS virus	–	LOD: 0.5 ng/mL < br > Sensitivity: 0.5 ng/mL to 5 μg/mL	High cost and potential for non-specific interactions	[65]
Sputtered gold film of 100 nm	CV	Sequence of SARS virus	–	LOD: 1 ng/mL < br > Sensitivity: 1 ng/mL to 10 μg/mL	Fragility and high cost of sputtered gold film	[66]
AMP with nanowire of In_2_O_3_	FET	Nucleocapsid protein of the SARS virus	–	LOD: 0.2 ng/mL < br > Sensitivity: 0.2 ng/mL to 2 μg/mL	High fabrication cost and potential for signal instability	[67]
Deposition of Au on the carbon electrode	CV	SARS virus sequence of 30 mer-	–	LOD: 0.5 ng/mL < br > Sensitivity: 0.5 ng/mL to 5 μg/mL	High cost and potential for non-specific binding	[68]
Au@Fe_3_O_4_ nanocomposites, Premix A preparation with Fe_3_O_4_ nanoparticles, and Premix B preparation with GO	Smartphone with DPV	SARS-2 RNA	–	LOD: 2 ng/mL < br > Sensitivity: 2 ng/mL to 20 μg/mL	Complexity in preparation and potential for interference from smartphone components	[69]
Carbon electrode with gold nanoparticles	SMWV	Human coronavirus, and MERS virus	–	LOD: 1 ng/mL < br > Sensitivity: 1 ng/mL to 10 μg/mL	High cost and potential for non-specific binding	[70]
Sheet of graphene	FET	Spike protein of SARS-2	–	LOD: 0.1 ng/mL < br > Sensitivity: 0.1 ng/mL to 10 μg/mL	Sensitivity to environmental conditions and potential for signal drift	[71]
Vero Cells membrane engineered	Bioelectric analysis	SARS-CoV-2	–	LOD: 0.2 ng/mL < br > Sensitivity: 0.2 ng/mL to 2 μg/mL	Complexity in preparation and potential for variability in cell membrane properties	[72]
AuNPs with fluorine-doped tin oxide	CV and DPV	nCovid-19 spike antigen	–	LOD: 1 ng/mL < br > Sensitivity: 1 ng/mL to 10 μg/mL	High cost and potential for non-specific interactions	[73]

These sensors can identify the analyte of interest, such as protein [74], nucleic acid [75], viruses [76], pathogenic bacteria [77], antibodies [78], carcinogens, and other biological molecules [79] on the surface of signal transducer [80]. The biosensing interaction layout is very important in confirming the efficiency of nano-biosensors [81]. Currently, researchers enhance the precision and sensitivity of the sensors by using nanocomposites and discovering the morphology of surface [82], such as nanoparticles [83], quantum dots [84, 85], nanowires [86], nanoroads [87], nanofilm [88], nanopillars [89], or carbon nanostructures [90]. Moreover, fabrication of nanostructure, microstructure, and pillars can have an excellent role in controlling and increasing the detection effect [91]. Several combinations allow these nano biosensors to perform various applications in therapeutic and ecological sectors owing to their direct response and recognition. One of the vital benefits of nano biosensors is their excellent efficiency in sensing microorganisms and viruses at very little concentration. These nanosensors are especially useful in urgent conditions to detect respirational diseases, including new coronaviruses SARS-2 virus [92].

In this study, we analyze the use of nanomaterial-based sensors for the detection of respiratory viruses, including coronaviruses. We review recent research on nano-biosensors to evaluate their efficiency and rapidity in uncovering and diagnosing respiratory viruses.

## FET-based nano-biosensors

FET-based nano-biosensors are a new technology merging the established world of field-effect transistors (FETs) with the exciting realm of nanomaterials. FETs are tiny electronic switches that can be influenced by their environment. In biosensors, this environment includes biological molecules. FET-based nano-biosensors take this a step further by incorporating nanomaterials like graphene or carbon nanotubes. These nanomaterials boast a massive surface area at the nanoscale, perfect for capturing and detecting miniscule amounts of biological molecules. This combination offers a powerful tool for highly sensitive detection of biomarkers and other biological targets.

Field effect transistor-based nano biosensors have many benefits and applications as compared to other strategies. These nano biosensors are extremely sensitive and show measurements instantly at very low concentrations of bioanalytics [93]. Field effect transistors-based nano biosensors are useful in several applications specifically in the medical field and diagnosis [94]. Graphene is a leading nanomaterial which consists of a 2D layer of carbon atoms. This is due to the admirable synthetic, physical, and electrical features of carbon nanomaterial [95]. Therefore, the combination of graphene-based installed nanomaterial can be a suitable applicant for field effect transistors-based biosensors [96].

A field effect transistor is a tool that consists of three terminals namely drain, gate, and source. In FET, an electric field is applied at the entryway terminal which customizes the transmission of electricity of the channel located among the source and drains [97]. Graphene is utilized as a channel in the FET structure. The excellent conductivity of graphene reduces the feedback duration of GFET which helps in the rapid detection of the virus [98]. The virus is stagnant on the graphene surface which alters the conduction of graphene which is immediately distinguished at the output [99].

Due to their reliability, downsized capability, and rapid detection ability with minimum cost and high output, GFET nano biosensors extensively work as POC diagnosis of several types of diseases [100]. Huge scope of GFET in biosensing, scientists have utilized contemporary displaying and simulation systems to increase the feedback of GFET biosensors [101]. Cahn et al. made a condensed graphene oxide transistor for boosting the sensing power of Bio-FET sensors for influenza H5N1 virus gene detection in a smooth environment [102]. Yu-jen Chen et al. developed a wireless graphene-based FET biosensor that is highly sensitive and bio-specific in detecting the virus H1N1 virus [103]. *Importance* Graphene-based FET nanobiosensors are considered as great space in biomedical diagnostic due to their high sensitivity, rapid point of care testing, affordability that makes them likely to succeed in this field.

### Advancement/ future potential

Residing at the crossroad where nanotechnology and biotechnology integrate is where GFET-based nano biosensors come to light which can be capable of massive improvisation in medical diagnostics. GFET biosensors are distinguished by the superb sensitivity, at the same time they can register biomarkers in extremely small amounts of which are a great importance for early-stage diagnosis. Also, graphenes' outstanding conductivity allows it to function within milliseconds after observation and therefore, may lead to swift virus identification as well as provide more timely treatment procedures.

GFET biosensor advantages such as high sensitivity, reliability, and selectivity due to the competence of test molecules to bind solely with the target polymers are the reasons that make them very important in the medical field. With their portable shape and the high-efficiency design, POC diagnostic tools are springing into fulfilling their mission at the distance as well as the scarce resources settings where they are ultimately needed.

The proceedings of this research are ongoing currently, in which there is an interest in methods to upgrade the GFET performance. Research such as those of Cahn et. al. and Y.J.Chen et. al. show this field's significant application in practical disease detection as GFETs can reach higher sensitivity and relativity to biochemicals than other current methods. Due to the thoroughness of research, GFET biosensors face a chance of prevailing over the current drawbacks and be supposed the highest level of specificity and speed of the medical screening tests [104–106].

### Limitations

FET bio sensors based on graphene permit a detection of diseases in minutes and with ultrasensitive features. Problems arise when such sensors need to be specific (target right molecule), have been functionalized and be scalable, or they simply become too costly for practical use due to fine-tuning of graphene properties to overcome the limitations (Debye length).

**Fig. 1:**
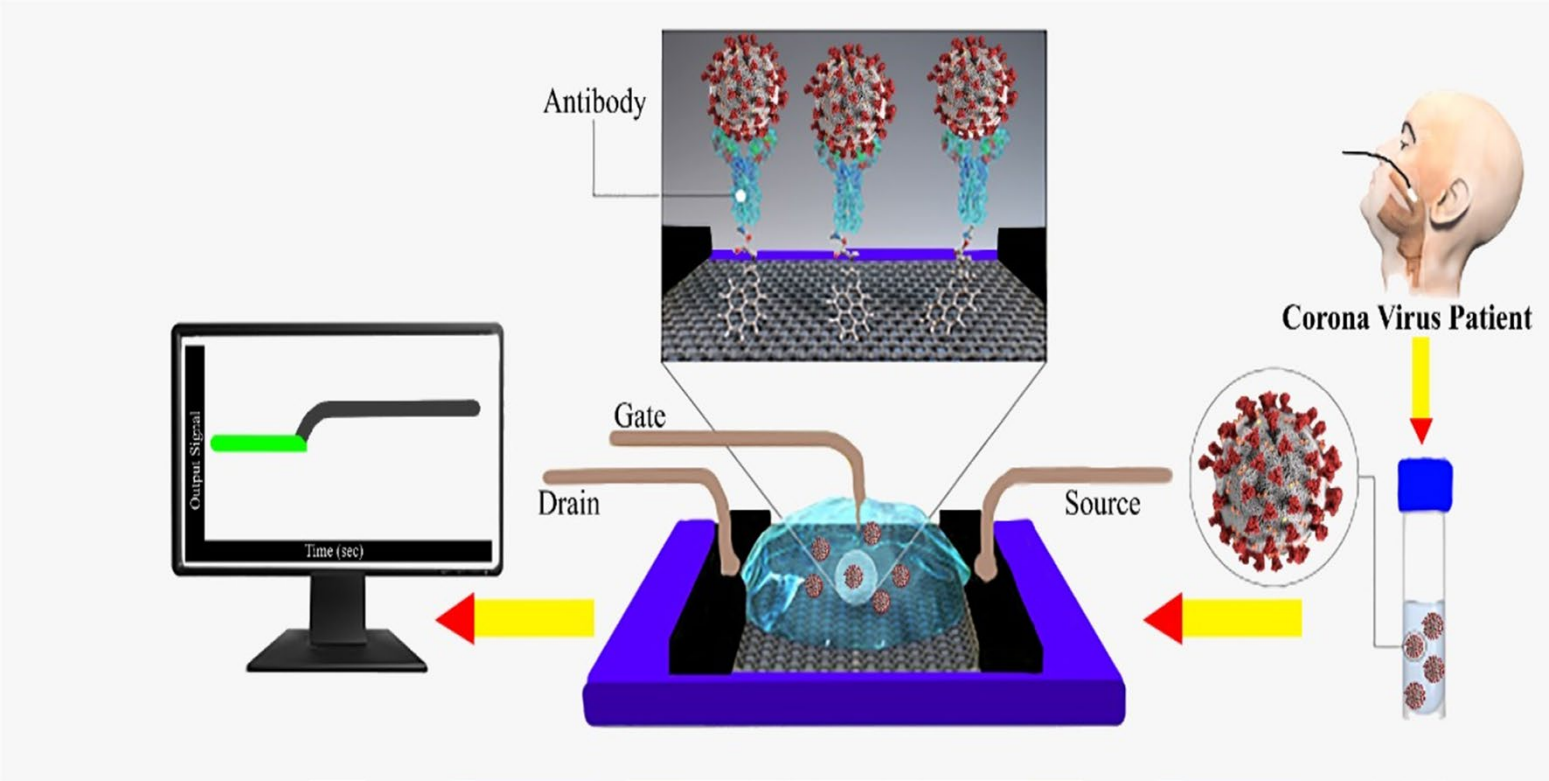
Schematic illustration of FET-based nano biosensors for rapid diagnosis of COVID-19

Economized and reproducible GFET sensors having excellent strength for unfailing analysis of the disease with great sensitivity are needed for the recognition of the early stage of SARS-2. Graphene is an amazing material thus it can be suggested that graphene will take over all the blockades in the upcoming days and GFET-like biosensors will be the extraordinary biosensing devices for graphene. Figure 1 demonstrates the rapid diagnosis of coronavirus by the field effect transistors-based nano biosensors.

## Plasmon resonance-based nanobiosensors

Plasmon resonance nanobiosensors emerged from the scientific understanding of surface plasmon resonance (SPR). SPR is a phenomenon observed when light interacts with metallic nanoparticles, causing their electrons to collectively oscillate. This oscillation resonates at a specific wavelength of light. The history of SPR can be traced back to early studies in the twentieth century, but it wasn't until the development of sophisticated nanotechnology that researchers began harnessing its potential for biodetection. By incorporating tiny metal nanoparticles, typically gold or silver, into biosensors, scientists unlocked a powerful tool. The size and shape of these nanoparticles influence the resonant wavelength, and when biological molecules bind to their surface, the surrounding environment changes. This change can be detected by monitoring the shift in the resonant wavelength, revealing the presence and potentially the quantity of the target biomolecule. This label-free detection method, relying solely on the interaction of light with the nanoparticles and the target molecule, forms the core principle of plasmon resonance nanobiosensors.

Biosensors based on plasmon resonance (SPR) have been studied widely [107]. Several designs of SPR constructed on prism [108], grating [109], visual fibers [110], couplers [111] and assimilated with optical wave guide [112] have been recognized and used for the analysis of biochemical and biotic measures [113]. Surface plasmon resonance (SPR) is a very suitable way for biomolecular dealings anatomization [114, 115]. In the bimolecular interaction analysis (BIA) SPR technique, one of the interacting molecules (antibody) is deactivated on the surface of the sensor [116, 117]. Whereas the added (antigen) is present in the fluid taster and taken close to the surface of the sensor, which permits the contact of biomolecules to take place [118]. SPR system is very sensitive, efficient feedback (retort period), label permitted, and the actual time of binding procedures among biomolecules and surface. The SPR method is beneficial for noticing the change at the nanoscale boundary very efficiently [119]. The SPR-based technique is widely used for biosensing-based detection of biomolecular analytes. Surface plasmon resonance (SPR) biosensors are utilized in many areas like biochemistry, applied science, and biomedical as investigative probes for transferable disease, protein-DNA acquaintances, ion sensing, binding actions, and biological surface alterations [37, 120–124]. Nur Alia Sheh Omar et al. created an Au/DSU/amine-functionalized rGO-PAMMA narrow film-based surface plasmon reverberation (SPR) gadget that was intended for the acknowledgment of DENV 2 E-proteins. The developed sensor showed excellent exposure accuracy, sensitivity values, good representation of quality, and gesture-to-sound ratio, up a growing shift in the SPR angle. Moreover, the designed sensor also depicted excellent results against DENV 2 E-proteins as compared to DENV 1 E-proteins and ZIKV E-proteins [125]. Nur Alia Sheh Omar et al. developed cadmium sulfide quantum dots composited with amine-functionalized graphene oxide (CdS-NH_2_GO) thin film-based surface plasmon resonance (SPR) sensor for dengue virus (DENV) E-protein. They also attached a special type of monoclonal antibody (IgM) with CdS-NH_2_GO through EDC/NHS coupling to detect targeted E-proteins. SPR sensor showed outstanding finding perimeter and sensitivity for the exposure of dengue virus E-proteins [126]. Joshua A. Jackman et al. developed an mPEG (polyethylene glycol)-SH-functionalized nanohole array-based surface plasmon resonance (SPR) sensor for dengue virus-like particles, which showed excellent detection against dengue virus-like particles [127]. Zhao et al. developed a self-assembled monolayer of isopropanol chemically on the gold surface of the optical fiber-based surface plasmon resonance (SPR) sensor through the plasmon modification method. The result depicted that SPR-based sensors can be utilized for the recognition of avian influenza subtype H6 quickly [128]. Similarly, SPR is also utilized for the finding of aerial pathogens. AuNPs-alloyed quaternary 1-cysteine-capped CdseTeS quantum dots were used for the analysis of H1N1 and H3N2 growing pathogens in 15–20 min [41]. The result showed excellent detection of H1N1 in deionized aqueous medium and human fluid. The suggested technique was also useful in the discovery of clinically isolated Norovirus and H3N2 [41]. Another study was conducted on the SPR-based fluorescence-enhancement mechanism for the detection of norovirus-like particles using CdSe-ZnS-based quantum dots. The assessment criteria of the considered instrument were 0.01 ng/mL [39]. *Importance* Biosensors involving the Surface Plasmon Resonance (SPR) have high sensitivity, they provide real-time monitoring as well as they are label-free detectors of diverse biomolecule interactions that are helpful in biochemistry, diagnosing diseases and environmental monitoring.

### Advancement/future potential

Though Surface Plasmon Resonance (SPR) are a useful instrument in biomolecular investigations, the biomolecular community is still on a rise and an array of cutting-edge advancements is vigorously being adopted that can change the future. Nanobiochsensors, specifically, open up a new line of possibilities compared with the regular methods that we are used to. The sensitivity in their unusual ability to detect tiny traces of marker molecules helps to reveal early diseases and monitor environmental factors more effectively. Additionally, nano sensors are adaptated which have faster reaction times thus providing decision making without delay. Additionally, to SPR, innovations including FET biosensors amalgamating graphene and nanoparticles ensure the future of more selective and prompt detections of biomolecules. The consequences of these technologies are very important, because they have the potential of revolutionizing during of the disease through early diagnoses, personalized medicine and improved monitoring of the process. Environmental protection can be essentially fostered by real-time pollutant detection nano biosensor system. Also, the quick identification of pollutants (contaminants) is a technique of increasing the security and wholesomeness of food. With time, as the research in medical nano sensors progresses, no doubt we will be witnessing the birth of many new groundbreaking findings that will have a significant effect on health care, environmental monitoring, and also other fields [104–106].

### Limitations

The SPR biosensors happen to be highly sensitive and at the same time versatile instruments for the bio dreams detecting. They have become an instrument that allows real-time visualization and confirmation of the bond formation between the molecules, this is the factor that makes them valuable in biochemistry, medicine, and environmental monitoring. But then, we can never settle for less. Although mini-SL AMR sensors are known to be sensitive, the barriers also exist. For example, analyzing exactly two similar molecules (high precision) becomes difficult once they begin to differ.

**Fig. 2:**
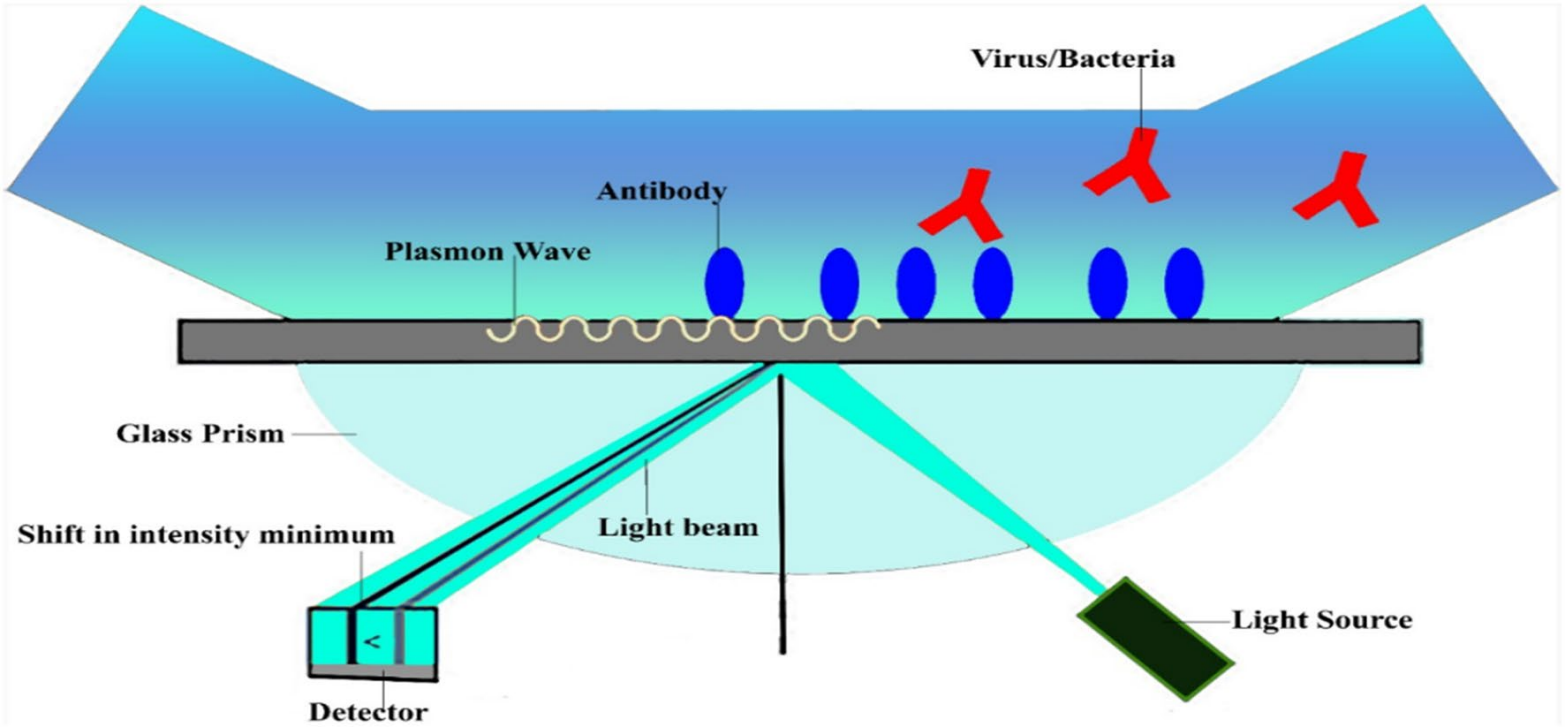
Schematic illustration of detection of viruses and bacteria through SPR-based nano biosensors

Figure 2 shows the analysis of viruses and bacteria through SPR-based nano biosensors.

## Surface-enhanced raman spectroscopy-based nano-biosensors

Surface-enhanced Raman spectroscopy (SERS) based nano-biosensors are a relatively recent innovation stemming from the established field of Raman spectroscopy. Raman spectroscopy itself has a rich history, dating back to the early twentieth century, where it analyzes the vibrational fingerprint of molecules. However, its application was limited due to weak signal generation. SERS revolutionized this by utilizing specially designed nanomaterials, often metallic nanoparticles like silver or gold. These nanostructures create intense localized electromagnetic fields when struck with light, dramatically enhancing the Raman signal of molecules adsorbed on their surface. This newfound sensitivity opened doors for SERS-based nano-biosensors. By attaching biorecognition molecules like antibodies to these nanostructured surfaces, scientists can create highly specific sensors that can detect miniscule amounts of targeted biomolecules with exceptional detail. This combination of nanotechnology and Raman spectroscopy paves the way for powerful bio-detection tools in various fields.

Raman Spectroscopy is used for the analysis of material components and depicts fingerprints excess of molecular elements. It is a very significant technique for biochemical study because it can be utilized efficiently for samples present in an aqueous medium and on active biological cells. To amplify the Raman signal uneven metal surfaces or nanometal particles are used, which made it easy to sense organic dyestuff in art and several viruses [129–132]. One advantage of using SERS that, it can detect even a single molecule [133]. SERS showed excellent properties in the detection of low-concentration biochemistry samples. Apart from outstanding sensitivity, SERS has also other applications in which fast measurement, nondestructive testing, and no sample pretreatment are included [134]. Two mechanisms are ascribed to SERS (i) Electromagnetic (EM) enhancement and (ii) Chemical enhancement [135]. EM enhancement is the most reliable method due to the interdependence between surface plasmon and incidental laser light at the metallic surface of the nanostructure, EM field intensity devolution takes place at the surrounding of the metal substrate which tremendously increases the excitation intensity of the Raman scattering process. SERS signals can be further increased when laser excitation wavelength corresponds with the wavelength of the local surface plasmon resonance (LSPR) of the substrate. The substrate nanostructure works as a nanoantenna to convey and increase Raman scattered light. Excellent substrate materials for SERS are noble metals like copper, silver, and gold because these noble metals perform a vital role in generating plasmon resonance in the visible and near-infrared range. Chemical enhancement is interconnected to elevate the transition polarizability of the analyte molecules when they arrest on the surface of the nanostructure. When the molecules are absorbed onto the metal surface then intermediary transfer of electronic charge takes place. This advanced electronic incorporated state behaves as a resonant state which activates an electronic resonance Raman enhancement. One of the other techniques for chemical enhancement includes holes and electrons which are produced in the metal and then combined into the molecular orbitals [136]. Chemical SERS enhancement is an activity that particularly occurs at adsorption places; these places are called active sites. When both the process (EM enhancement and chemical enhancement) outcomes are taken into description, the enhancement level is theoretically believed to be on the order of 10^14^ [137]. Lei Zhan et al. used peroxidase substrate 3, 3’-5, 5’- tetra- methylbenbenzidine (TMB) as a Raman molecule for the recognition of respiratory syncytial virus. Oxidation of TMB by horseradish peroxide leads to the initiation of TMB^+^. These positively charged ions of TMB are attached electrostatically to the negatively charged outer surface of AgNPs. The Stockpile of AgNPS is the source of generating excellent SERS signals. The lowest limit detection of SERS dependent enzyme catalyzed immunoassay was 0.05 pg/mL which is 20 times less than that established in the colorimetric system [38]. *Importance* SERS (surface-enhanced Raman spectroscopy) provides a sensitivity level as low as single molecule and thus it is a really strong tool for elucidation of biomolecules in liquid environments and within living cells.

### Advancement/future potential

Nanobiosensors is a booming sector of science with individual techniques that stand out. Surface Enhanced Raman Spectroscopy (SERS) technology is one of the most sensitive. In comparison to the aforementioned classical Raman spectroscopy, SERS is able to detect molecules even at very small quantities, making it a point to analyze of tiny proteins in lower concentration fluid samples. Thus, more illnesses will be diagnosed in an earlier stage, and the situation of environmental monitoring will be more active and effective. Beyond high sensitivity, SERS provides diagnostics with multi-features such as speed, destructivity-free testing, and minimized sample preparation, thus transforming the testing process as a whole. The essence of this magnifying strategy comprises two megaphones—one of which is called signal amplification. The enhancement based on electromagnetic (EM) interaction is an integral part of Raman spectroscopy analyzing process. The reaction between surface plasmons and the laser light on the metallic nanostructure produces another laser wave which is much more powerful compared to one in the initial state. Chemical enhancement attains a chemical enhancement state whereby the molecules of the analyte bind to the nanomaterial and a given electron charge is then transferred to the structure, leading to an intensified signal. The effect of the quantum combined technique is multiplication of factors at a theoretical level to the maximum value of 10^14. The most eminent capability of SERS is demonstrated by Lei Zhan and his team with their research that enabled a detection limit of 0.05 pg/mL for a specific virus via SERS-based immunoassay that is capable of outperforming traditional methods with the ratio of 20 to 1. Such outstanding sensitivity and multiple possibilities are the strengths that are already used in the development of the next-generation nano structors, which can be a tool for researchers, and probably by revolutionizing healthcare, environmental monitoring and nearly countless other fields [104–106].

### Limitations

Subsequently, SERS can be used for material analysis on the molecular level with the high power of sensitivity. In addition to that, its features such as water-compatibility and the ability to be used with living cells are the ones which make it stand out among other imaging methods of today. The real magic is that SERS is not just able to detect millions of atoms, it can actually detect single molecules! This property is the most prevalent when working with low-concentrations samples. On one hand, needs no advanced hackery and doesn't destroy the sample, which makes its usage more convenient.

**Fig. 3:**
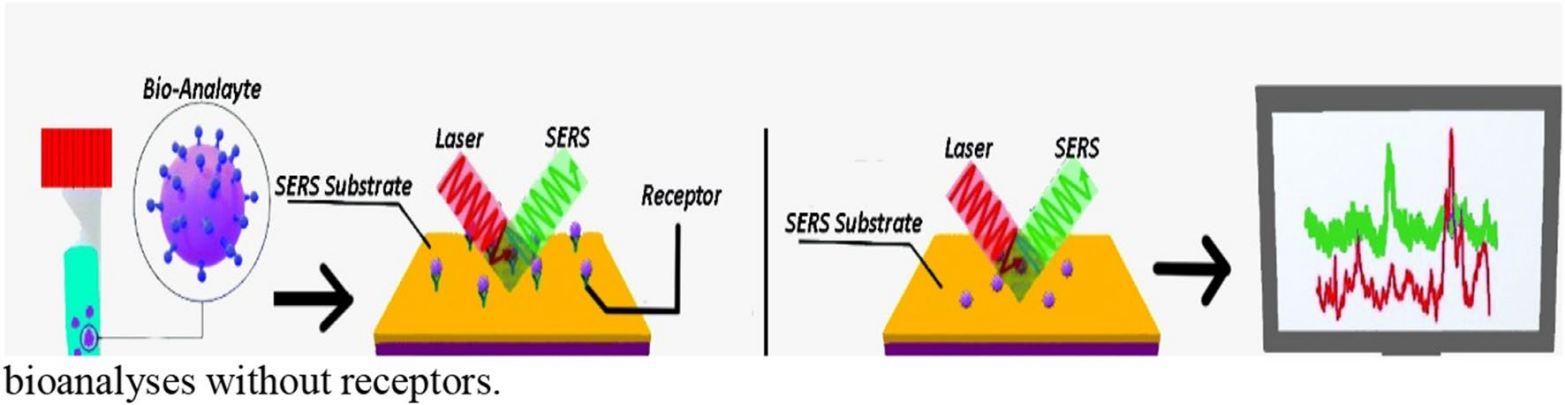
**a** Schematic Illustration of detection of Bio analytes through label-based Surface-enhanced Raman spectroscopy, **b** Detection of bio analyte through label-free surface-enhanced Raman Spectroscopy

Figure 3a shows receptors-based detection of bio analytes and Fig. 3b shows detection of bioanalyses without receptors.

## Electrochemical impedance spectroscopy (EIS) based nano biosensors

Electrochemical impedance spectroscopy (EIS) based nanobiosensors are a product of merging two well-established techniques: electrochemistry and nanotechnology. Electrochemistry, with a long history dating back to the eighteenth century, studies the relationship between electrical current and chemical reactions at an electrode's surface. EIS, a subfield of electrochemistry, goes a step further by analyzing the impedance, a combined measure of resistance and capacitance, of an electrochemical system across a range of frequencies. Nanotechnology, on the other hand, deals with the manipulation of materials at the nanoscale. In EIS-based nanobiosensors, scientists leverage the unique properties of nanomaterials to modify the surface of electrodes. These nanomaterials, like carbon nanotubes or metal nanoparticles, offer a significantly increased surface area compared to traditional electrodes. This translates to a greater number of biorecognition molecules, such as antibodies, that can be immobilized on the sensor surface. When a target biomolecule binds to these receptors, it alters the electrical properties of the electrode–electrolyte interface. By measuring the changes in impedance through EIS, scientists can detect the presence and potentially the quantity of the target biomolecule with high sensitivity. This combination of electrochemistry and nanotechnology has opened doors for a new generation of powerful and versatile biosensors.

The electrical impedance spectroscopy (EIS) system is a useful technique for bacteriological assessment, rust checking, a quality check of coating and cement fixative, depiction of compact electrolytes, and examination of human physique [138]. Recognition of germs through Voltic evaluation was of attention in 1898 when G. N. Stewart revealed the alteration in the development of contagious residents alters the conduction of growing medium [139]. The complete potential of the impedance microbiology technique was explained by many researchers in the late 1970s. This technique exploits the phenomenon of bacterial metabolism changes weakly charged or uncharged composites in highly charged compounds; thus, it fluctuates the electric characteristics of an expanding environment [138]. EIS technique is utilized to demonstrate biosensors and binding procedures on improved electrode surfaces such as DNA synthesis and antigen antibodies as well as to analyze tiny particles by checking the alteration in charge transmission impedance [140]. Electrochemical arrangements, identified as oxidation–reduction electrodes generally need a reference electrode and cathode, a working probe, and a counter auxiliary rod [141]. Over a period, the occurrence of transmittable diseases produced by the germs, such as Avian influenza virus, HPV, hepatitis virus, DENV, rabies virus (RABV), ZIKV, Human Virus, Chikungunya virus (CHIKV), HIV, enterovirus and the coronavirus has increased [142]. Coronavirus must be analyzed in the primary phases. Due to the characteristics of EIS, including low LOD, quick response, cost-effectiveness, unlabeled detection, and actual time analysis of the sample, this way can have a possible outcome for coronavirus finding. Recognition of pathological RNA via silica probe material draped with efficient graphene was distinguished. Pathological RNA was used to immobilize Deoxyribonucleic acid definite primers for hybridizing. Moreover, the EIS test determined important variances in surface R_ct_ on the noncomplementary DAN interbreeding and the complementary DNA interbreeding. Also, by amending this RNA recognition display place, the design of precise coronavirus DNA readers/primers, a definite grouping among probe (DNA primers) and the spike surface glycoprotein (S) /small envelop protein (E)/matrix protein (M) or nucleocapsid protein (N), can perform a central role in the judgment/analysis of COVID 19 [142]. *Importance* Electrical Impedance Spectroscopy (EIS) possesses the capability for different types of cases like speedy pathogen detection in Coronavirus isolation.

### Advancement/future potential

The domain of nano biosensors houses many latest inventions of diagnostic industry for health care and EIS stands fervently with its detection of bacteria in a real quick way. In contrast to the traditional methods, EIS capitalizing on the electrical changes emerging from the bacterial development makes it possible to directly track the bacterial cultures in real time without any labels. This means readier disclosure of bacterial infection, which may in turn be followed by the adaptation of better treatment methods.

EIS boasts several advantages that make it well-suited for use in nano biosensors:

- *Highly Sensitive* EIS can detect minute changes in bacterial activity, allowing for earlier infection detection.
- *Rapid Results* EIS provides quick turnaround times, crucial for making timely decisions in critical situations.
- *Cost-Effective* Compared to some traditional methods, EIS offers a more economical approach to diagnostics.
- *Label-Free Detection* EIS eliminates the need for additional labeling agents, simplifying the testing process.
- *Real-Time Monitoring* EIS allows for continuous observation of bacterial growth, providing valuable insights into infection progression. This technology holds particular promise in the fight against COVID-19.

Research states that these model-based sensors are made accordingly to the changes made by the surface resistance after the probes bind with the virus' RNA. Given that the design of probe can be optimized, and limitations can be adressed, EIS approach can be the most powerful alternative solution in the fight against COVD-10 and other infectious diseases. Its rapid, cheap, and at-the-site diagnostics process structure particularly in resource-challenged places make it suitable for such purpose [104–106].

### Limitations

EIS is an indisputably useful tool that serves a whole variety of different applications, but it naturally has some restrictions. The interpretation of information is another issue. It is not too easy to correctly implement EIS assay, which necessitates appropriate skills and competence in system utilization. As well, the electrode surface properties and the solution conditions (the movement of ions and the presence of waste products) can vary, so standardizing the procedure is a challenge at times. Also, it might be challenging for EIS to select a particular biological target that differs from others, which may produce inaccurate identification. Nowadays, researchers are involved in the philosophy of data interpretation, standardization of strategies, and identifying EIS, and also there is a focus on practicing these procedures for even more specific applications that are more reliable.

**Fig. 4:**
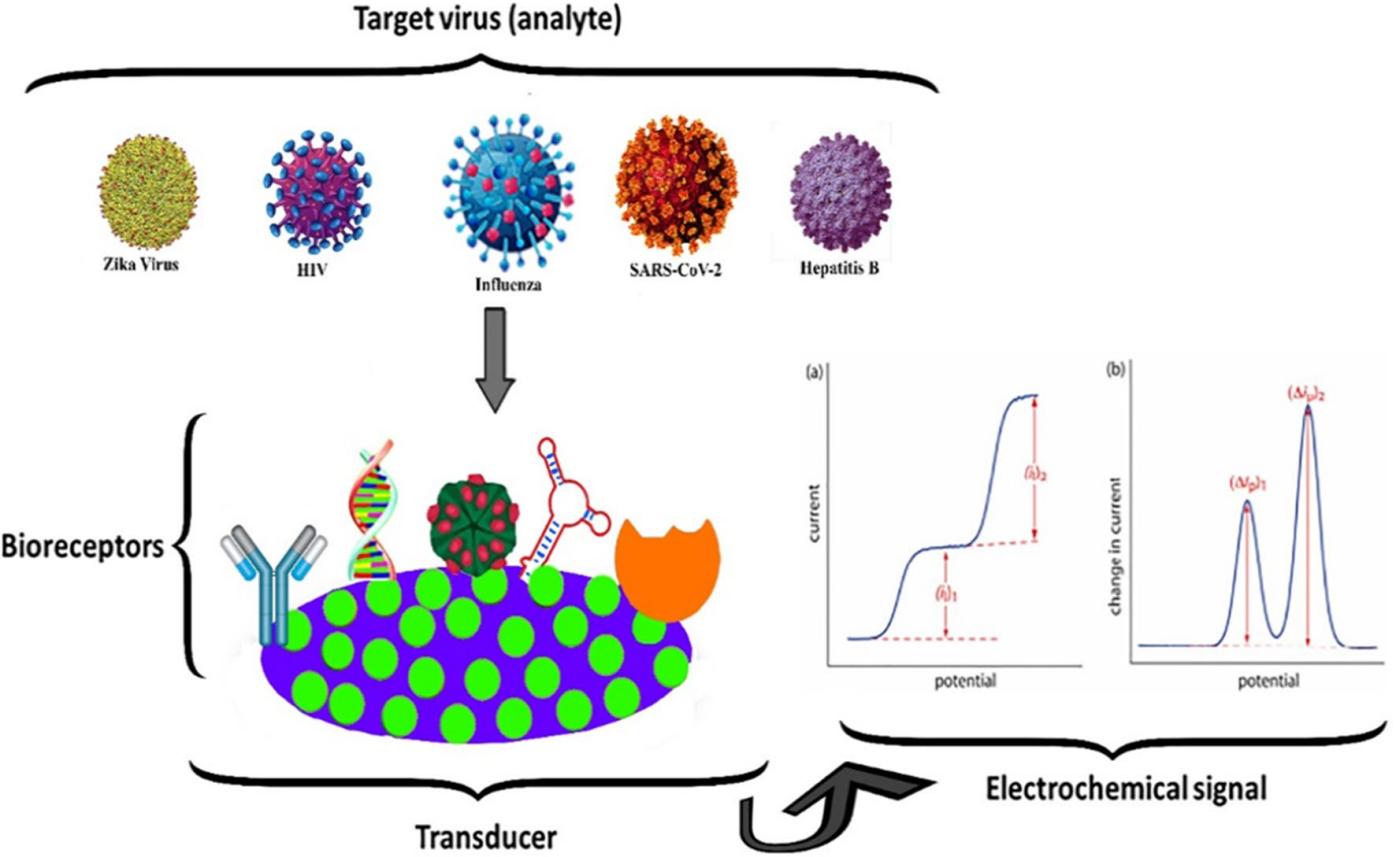
Schematic detection of Zika Virus, HIV, Influenza, SARS-COV-2, and Hepatitis B by Electrochemical Impedance based nano biosensors

Figure 4 explains the simple phenomenon of the detection of different viruses by electrochemical impedance spectroscopy-based nano biosensors.

## Metal-enhanced fluorescence-based nano-biosensors

Metal enhanced fluorescence-based nano-biosensors are a recent advancement that combines the established field of fluorescence spectroscopy with the unique properties of metallic nanostructures. Fluorescence, discovered in the late nineteenth century, involves the emission of light by certain molecules after absorbing light energy. However, the inherent weakness of this emitted light has limited its sensitivity in biodetection applications. Metal enhanced fluorescence (MEF) emerged as a solution. It utilizes the interaction between light and metallic nanomaterials, like gold or silver nanoparticles. When placed near a fluorophore (a fluorescent molecule), these nanoparticles can enhance the emitted fluorescence signal. This amplification allows for the detection of smaller quantities of the target biomolecule. The marriage of MEF with nanotechnology in biosensors unlocks a powerful tool for highly sensitive detection of biological markers and other targets. While the concept of fluorescence has been around for a while, MEF and its application in nano-biosensors represent a novel approach to biodetection with increased sensitivity.

A wide assortment of biological and chemical investigations depend on the utilization of fluorescence for describing a particular molecular action [143–145]. Signal strength is often a challenge and later attention to the methods that generate improved fluorescence signals, such as metal-enhanced fluorescence is deeply studied [146, 147]. MEF is a physical consequence that happens when fluorophores are positioned in nanometric space from the metal's outer surface. This method is distinguished by increased voluntary emission rate, photostability, and quantum yield, reduced fluorescent period of fluorophores, and may also be conducted by direct emission [146–148]. Fluorescence improvement variables of up to 500 have been accounted for hitherto in the confined mark of extraordinarily planned metal nanomaterial [149]. This enhancement aspect, along with the chance of direct outflow, has drawn in the consideration of the scientific research community because of the chance of identifying substances at very minimum concentration. The EMF effect also termed as plasmon-enhanced fluorescence [150, 151], surface enhanced fluorescence [152] and metal induced fluorescence enhancement [153, 154].

Such fluorophores can be combined with the metal in different ways: (1) energy transfer to metal from fluorophore; (2) concentration of electromagnetic field; (3) alteration of the radiative deterioration pace of the fluorophore by the ordinary alteration of the photon thickness of states. The primary approach, energy transmission, is a non-radiative interaction that overwhelms little space since this is conversely relative to the third force of the metal-fluorophore gap [146]. This is because of a connection between the fluorophore and the plasmon polariton at the outer layer of the metal [155].

Another approach, increasing the electromagnetic field is a standard physics effect, precisely defined by explaining Maxwell’s equations around the metal construction. For this situation, it has been observed that the electric field is increased around sharp corners/tips of metal nanomaterials, which is otherwise called “receiving wire" impact. In unambiguous frequency ranges, there is likewise the chance of resonances in the metal designs themselves. In metal nanoparticles, these happen in the noticeable close to IR frequency range, encouraging the formation of dipoles and increasing order modes [156]. The third component, the change of the radiative rate (I), is expected to increase photonic mode concentration near metal nanostructures [146, 147]. *Importance* MEF (Metal-Enhanced Fluorescence) has great potential the distinguish and detect biological and chemical materials with the highest sensitivity at exceedingly small concentrations.

### Advancement/future potential

The journey for more sensitive nano biosensors has been the bedrock for what can only be termed the exciting creation of metal–metal-enhanced fluorescence (MEF). This method consequently substantially amplifies the fluorescent signals of the probes commonly utilized in biosensors making them highly sensitive and capable of detection of the trace level of target analytes. MEF represents a tool with the ability to amplify fluorescent signals by up to 50 times, therefore, making it possible to detect even microscopic traces of relevant biological markers having a relation with diseases or environmental contaminants. It supplies not only heightened sensitivity but also additional options. It elevates the photostability of fluorescent probes which might prolong their lifetimes and decrease the amount of background noise. Consequently, MEF is more precise in identifying pathogenic components since the fluorophores are brighter producing high emission levels.

The magic behind MEF lies in the proximity between the fluorescent probes and a metal surface. This interaction triggers three key effects:

- Energy transfer from the excited fluorophore to the metal surface, further amplifying the signal.
- Concentration of the surrounding electromagnetic field by the metal surface, leading to stronger excitation of the fluorophore. This effect is particularly pronounced at sharp edges and corners of the metal nanostructure.
- Modification of the radiative decay rate of the fluorophore, resulting in faster emission of light and brighter signals.

Luminescence effects introduced to these systems make MEF a great tool for the creation of sensors with high sensitivity and also response rate. The capabilities of this technology are almost unlimited. By diagnosing disease, monitoring the environment, and building diagnostics points in remote and resource-limited areas, this technology can provide faster and more accurate results than conventional methods. Using MEF technology for biosensing not only allows to discovery of exceptional biomolecules at an amount of a trace but also may initiate revolutionary changes in the biomedical field, clinical diagnostics, environmental monitoring, and many other health and living conditions [104–106].

### Limitations

Although the presence of Metal-Enhanced Fluorescence (MEF) is a revolution in detection that magnifies fluorescence signals, it has deficiencies, therefore, limiting its wider application. It is still a challenge to understand how the different factors (electric field and magnetic field intensification, radiative rates) interact with each other in any circumstance. Specificity again is a problem, as MEF would not be exempt from the undesired molecules and molecules in view. Continued investigation into metal nanoparticles' design optimization to achieve consistency, as well as minimization of temperature contraindications in solution, is one of the pressing issues of modern research. The tailoring of these constraints builds confidence in the MEF which will now be a stronger scientific instrument for making discoveries.

**Fig. 5:**
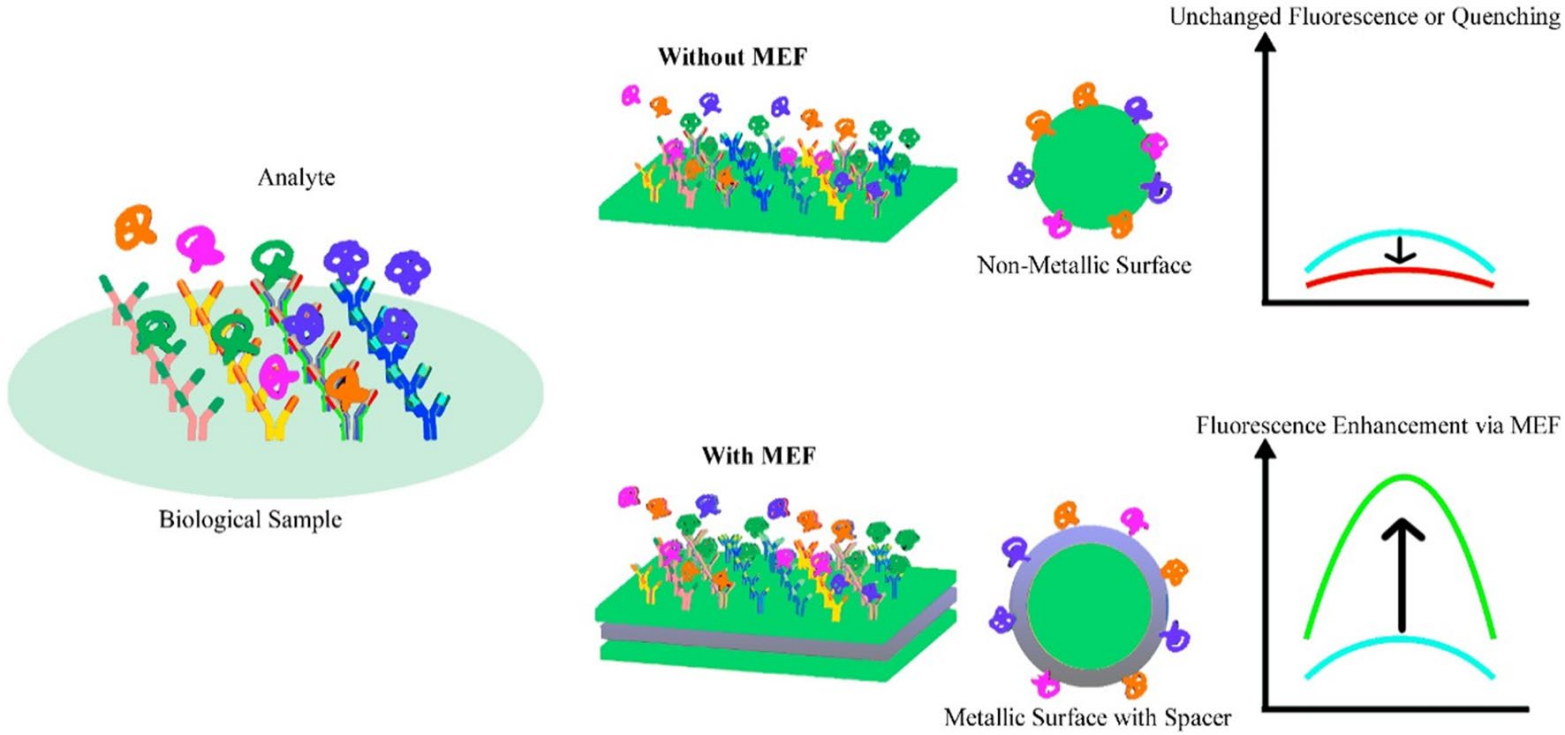
Detection phenomenon of different bio analytes with metal fluorescence and without metal

Figure 5 explains the analysis of various bioanalytics using metal and without metal-based MEF nano biosensors.

## Colorimetric based nano-biosensors

Colorimetric-based nano-biosensors are a fascinating blend of old and new. The core principle, detecting biomolecules through color changes, has been a cornerstone of analytical chemistry for centuries. However, recent decades have witnessed a surge in this technique thanks to nanotechnology. Traditionally, colorimetric assays relied on chemical reactions that produced colored products, but limitations existed in sensitivity and specificity. The introduction of nanomaterials, such as metallic nanoparticles or specific nanocarriers, has transformed this field. These nanomaterials boast a high surface area, allowing for the attachment of more biorecognition molecules like antibodies or enzymes. Additionally, some nanomaterials can amplify the colorimetric signal produced during biorecognition, leading to a more dramatic color change. This marriage of established colorimetric principles with cutting-edge nanotechnology has resulted in a simple, visual method for detecting biological targets, making it a valuable tool for various bio-detection applications.

In the coming days, sensing is based on many important factors such as economics, simplicity, and fast analysis. Sensors considering the colorimetric approach are very important in the assessment of model characteristics. Prior devices are uncaring to be mass and multifaceted, demanding, unlike efficient blocks such as analyzing units, finding units processing units, etc., which were responsible for delaying the response of sensors. Current innovation given colorimetry is about the scaling down of size, cost, in-situ, and with no extra tools. A calorimetric sensor is utilized for the momentary location of a substance and displays a variety of color variations, which can be seen by the naked eye easily. Nanotechnology plays a significant part in existing sensor innovation. For instance, nanomaterials like Cu, Ag, and Au are widely utilized in graphic analysis which is attributable to its visual property known as Surface Plasmon Resonance. Resonance plays a central role in the oscillation of free electrons with the incident light in the visible region, which is also known as the process of surface plasmon resonance [157]. Plasmon resonance scattering of Ag and Au nanomaterials has been applied for bio-affinity analysis [158]. Usually, the identification process depends on molecular contact on the outer layer of the substrate which is adjusted with nanoparticles or with some functional group [107, 157, 158]. The job of nanomaterials in colorimetric detection is accounted and their discussion has revealed that label-free evaluation is also conceivable with nanomaterials [159].

There are many difficulties engaged in fostering a powerful sensor, an ideal sensor ought to fulfill specific qualities like selectivity, responsiveness, robustness, exactness, accuracy, insignificant mistakes, reproducibility, linearity, etc. The sensitivity of a sensor is meant to be the feature to diagnose the analyte of attention from many other interfering models. The quality of a sensor to recognize the analyte indeed, even at extremely low focus is named as sensitivity. However, the ongoing innovation fulfills the previously mentioned difficulties. Lab-on-chip is one of the unmistakable stages in which sensor innovation is inferred with an elevated degree of achievement [160]. It includes basic and convenient gadgets made of polydimethylsiloxane (PDMS) being utilized for analyte analysis by streaming fluid samples inside a microchannel [160]. Microfluidics has acquired wide acknowledgment in sensor advances because of its low footprint and fewer users of analyte-containing reagents. Lab-on-chip innovation utilizing paper, for example, lab-on-paper (LOP) became conspicuous for its minimal expense, quick recognition, and self-supportability. Sensor platforms given LOP for the recognition of various biomolecules have previously been accounted for by Whitesides [161]. LOP is basic, modest, and effectively disposable. LOP utilizes cellulose paper for capturing the particles in a designated site and the identification depends on the colorimetric approach. Microarray utilizing LOP can recognize various examples all the while. *Importance* Colorimetric sensors are well-known for being low-cost, a simple way of analysis and also fast in use; that is why they are mainly applied to portable point-of-care diagnostic kits.

### Advancement/future potential

Tomorrow’s biosensors will all be sensible, quick, and cost-saving. The colorimetric biosensors (i.e. the sensors which detect and quantify the analyte colorimetrically) are the first choice in the ever-moving field of this trend. Even the eyes can determine the outcomes without special tools. The pod is kept after the seeds germinate, and the entire process is monitored by students, along with the dissemination of carbon dioxide and moisture in the air. Additionally, the production and operational costs of renewable energies is low compared to other sources that cost a lot thus, they are affordable and can be used effectively. Therefore, the main advantage of the colorimeters lies in the speed of processing results, which can up to speed decision-making process.

Nevertheless, colorimetric sensors can be improved thanks to nanotechnology which is considered to be one of the hottest fields of science and technology today. For this purpose, a few aged gold or silver nanoparticles are taken, increased concentration of these nanoparticles are used and they have a brilliant property of a glance called Surface Plasmon Resonance (SPR). Light can cause these nanobodies to give off a striking palette of colors when it runs across them. The capacity to enhance the detection signal by an order of X0 is explored to reach the point of detection of target molecules that are in the least detectable concentrations. As evident, simplicity is indeed a strong aspect of the sensor design but researchers address other crucial aspects as well. Selectivity is increasingly becoming better, i.e. they becoming more title to discriminate among the target molecule and other substances. Sensitivity, the realization of substances even in a small amount, is another area that technology is focused on. For the last of it, the people are working on the quality and result, that is, for the same test, at different times and with different test kits, they expect to get the same result.

LOC technology reduces this artificial color-making application to the smallest chip, thus making it more operational and precise. These microfluidic devices incorporate small qualitative analyzers using microchannels which are employed in capturing the samples quickly and in an organized manner. Also, the most interesting development of the Laboratory-on-Paper (LOP) technology is worth mentioning. That is to say that these disposable sensors made of paper use very little money and are perfect in remote settings as well. This platform proves to be interactive and convenient as it was developed for colorimetric detection of multiple biomolecules [104–106].

### Limitations

Colorimetric sensors are feature-rich with their low cost, convenience in operation and also quick response times. However, limitations remain. Another feasibility lies in the high concentration in the composition of detections of the target molecule, ignoring other interference subsumption, that can cause false positive results. Another obstacle is in the sensing part, due to their limited sensitivity such colorimetric sensors could not be used to determine very small concentrations. In summary, flow meters and metering devices mentioned above have linear response and offer considerable advantages such as being easy to use, maintenance free, reliable, and accurate. However, the colorimetric sensors are the most flexible but also the most expensive ones. Researchers developing the most specific, sensitive,and reusable colorimetric sensors are the major focus of their effort to make them even more dependable and friendly to users.

**Fig. 6:**
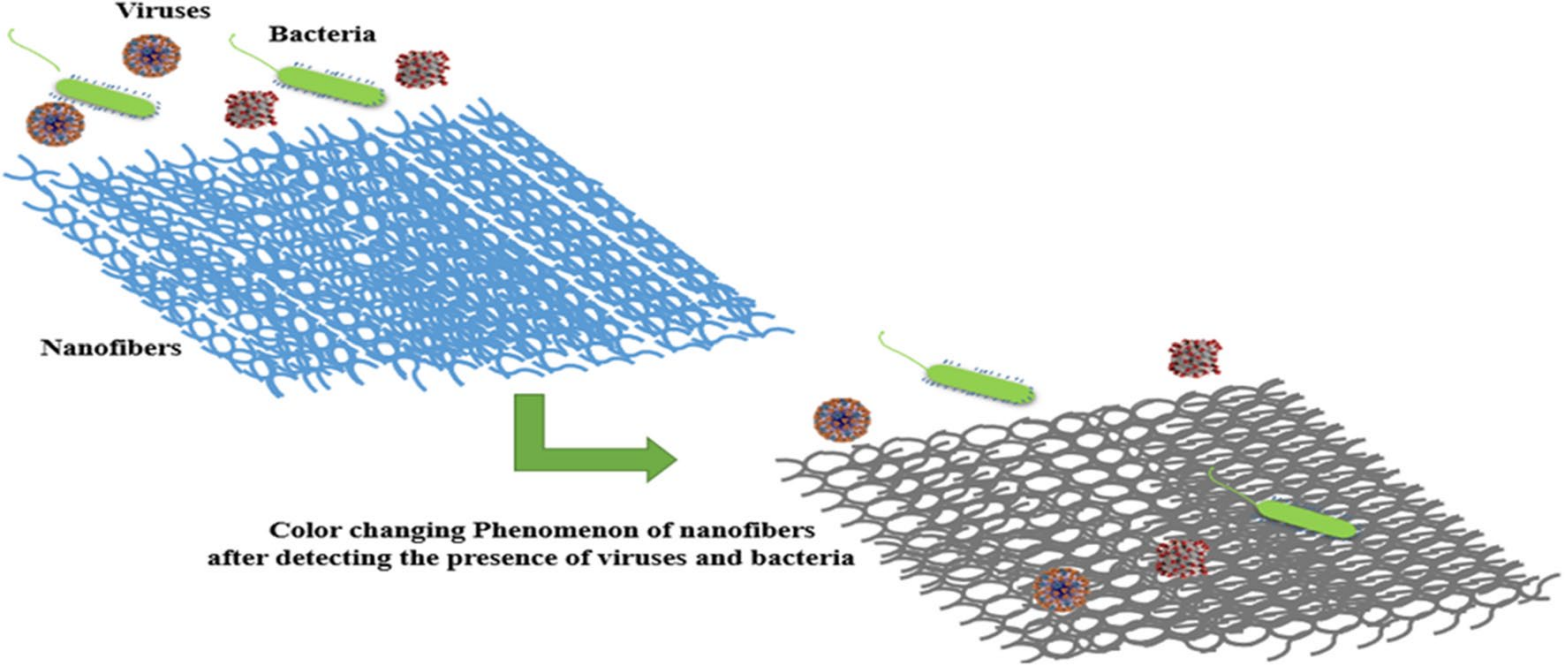
Schematic illustration of the color-changing phenomenon of colorimetric nano biosensors after detecting viruses and bacteria

Figure 6 shows colorimetric-based nano biosensors that change color after detecting viruses and bacteria.

## Case studies

### Colorimetric-based nano-biosensors

COVID-19 Diagnostics: Colorimetric nano-biosensors have developed for specific rapid response to COVID-19 diagnostic. The scientists invented a detector that changes its color from green to red when SARS-CoV-2 RNA is present and gives a color reading in less than an hour.

Food Safety Monitoring: Nanobiosensors which are based on colorimetry are one of the recent methods that have been employed for food examination, particularly in detecting contaminants such as pesticides or toxins. Instance, surprisingly, the indicator that changes color when it meets with organic phosphorous pesticides can be hopefully used for monitoring food safety.

### SERS-based nano-biosensors

Bacterial Detection in Food: Through SERS biosensors that respond to fother devices, researchers have been able to detect Salmonella rapidly in food samples. Biosensors relied on salmonella-specific antibodies encapsulated with gold nanoparticles. The biosensor can detect and show results most of the time in under an hour.

Drug Monitoring in Chemotherapy: SERS-based nano-biosensors have been employed and patients' blood levels of doxorubicin have been monitored in many recent disease trials. The sensors employ gold quanta box nanostars to obtain exquisite and fast recognition of the drug with higher accuracy which will lead to better drug delivery.

### FET-based nano-biosensors

COVID-19 Detection: Scientists built a field-effect transistor-based nano-biosensor as the means of locating SARS-CoV-2 in persons' saliva. The biosensor applies graphene layers whose antigenic receptors are attached to the spike protein of the coronavirus. This machine was able to deliver quick and sensitive detection of COVID-19 but in a not intrusive nature.

## Future directions

**Fig. 7:**
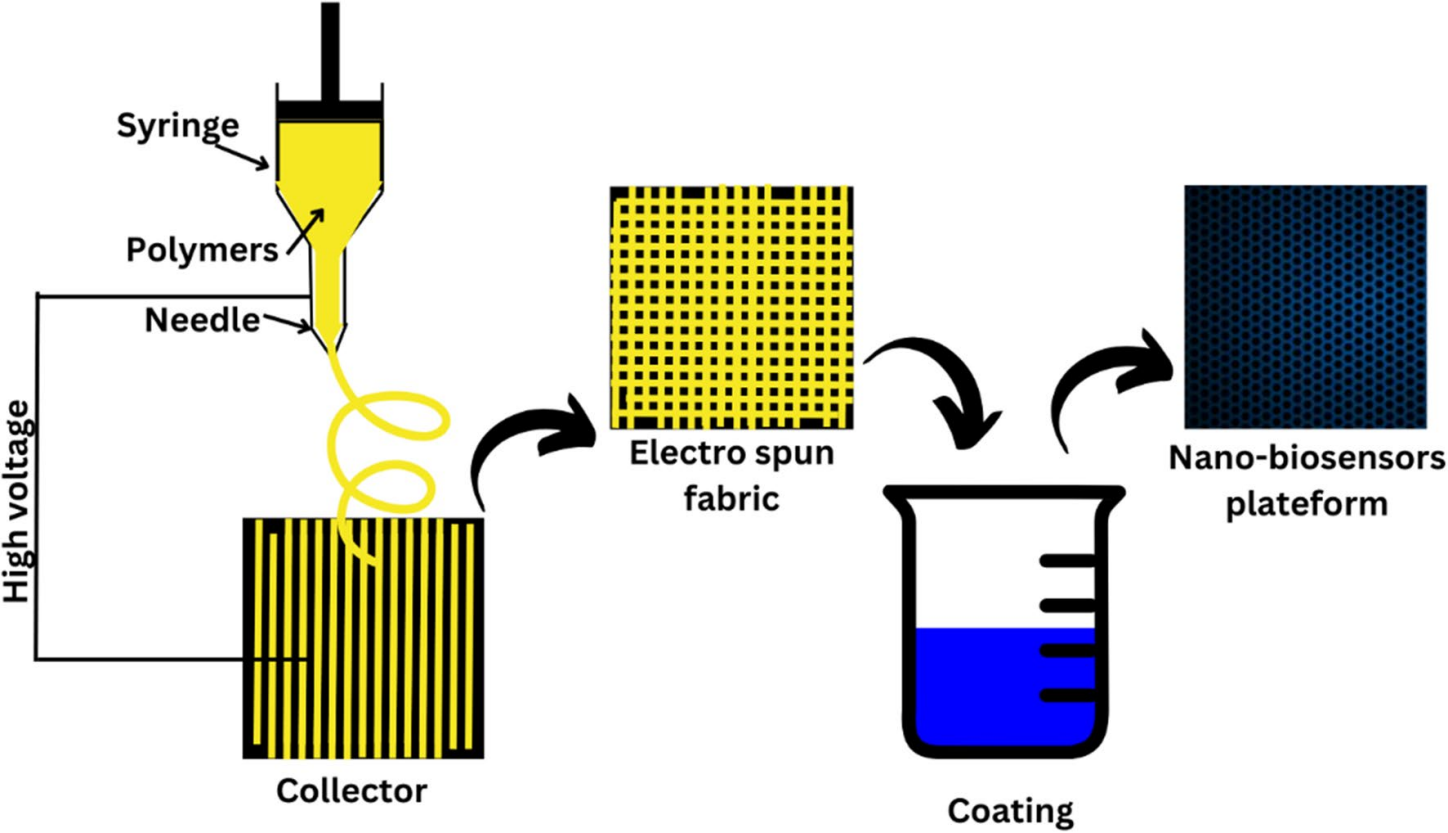
Electrospinning of nano-biosensor fabric

**Fig. 8:**
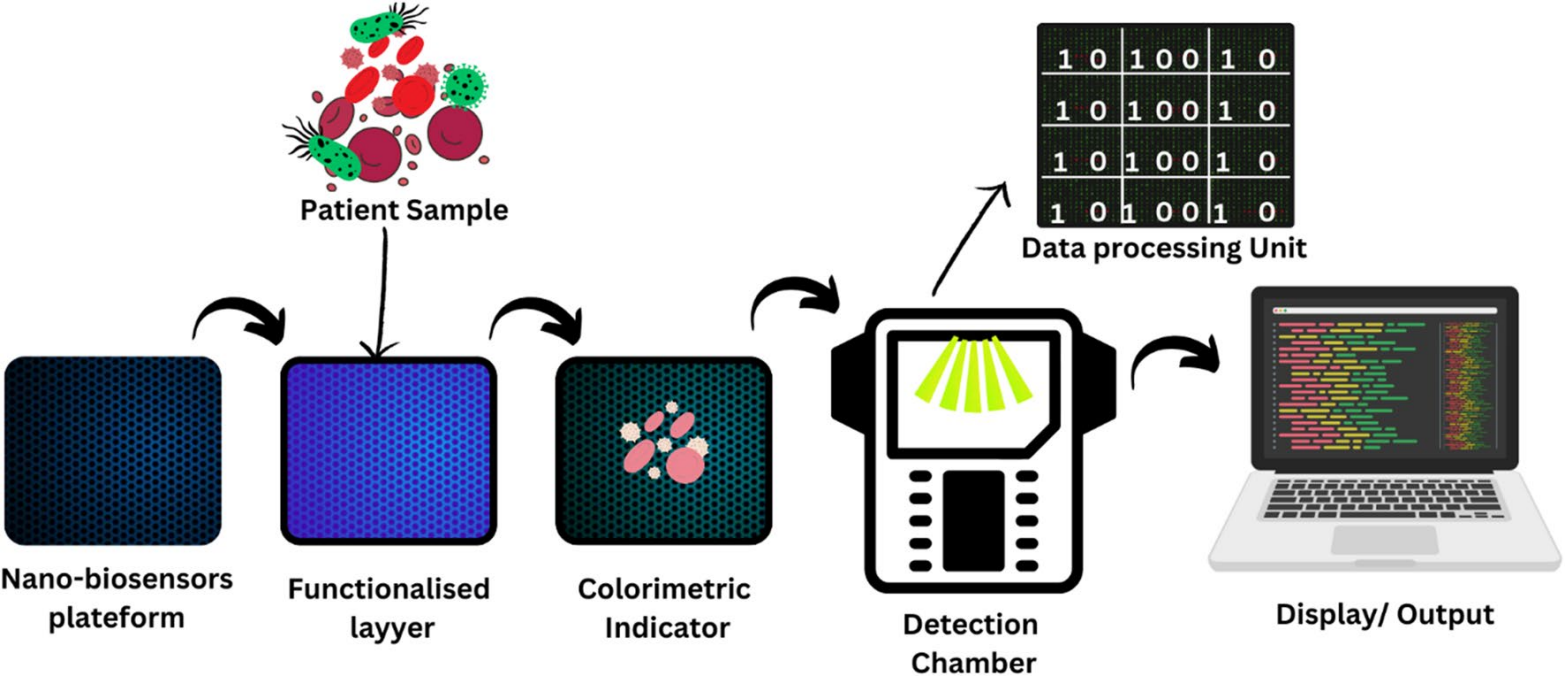
Colorimetric nano biosensor: A rapid and visual approach for targeted virus and bacteria detection

Nvestigate how to include cutting-edge artificial intelligence algorithms to increase the precision and speed of airborne microbe identification developed by electrospinning as mentioned in Figs. 7 and 8. By using machine learning methods for data analysis, we can enable the nano-biosensors to adjust and enhance their functionality over time. It would be possible to create intelligent algorithms that can recognize patterns in intricate datasets and make decisions about the detection of microorganisms in real-time.

Examine and use new nanomaterials with special qualities for the creation of biosensors. To improve the sensitivity and specificity of nano-biosensors and allow for the detection of a wider variety of airborne microorganisms, we ought to concentrate on surface changes. To increase the adaptability of the biosensor platform, experiments using multifunctional nanomaterials that can interact with different microbes at the same time could be conducted.

Improve lab-on-a-chip downsizing methods to make technology more movable and intuitive. To enable adoption in settings with limited resources, emphasize the development of affordable lab-on-paper solutions. Examine methods to improve lab-on-a-chip technologies' self-sustainability so that accurate and effective detection can be achieved without the need for complex infrastructure or external power sources.

## Conclusions

Over the most recent time of many years, viral and bacterial microorganisms have turned into a genuine hazard to human security. Their fast identification should be considered as an important undertaking to predict an occurrence, which implies a high liability of interruption of the medical care framework, and a lamentable financial influence. Researchers are conducting escalated analysis for creating delicate symptomatic strategies and powerful therapeutics. There is no immunization or pharmacological treatment for some infections and microorganisms and the expansion of POC strategies for the speedy analysis of viruses such as COVID-19 tolerates fast-tracking lifesaving decisions, and isolation of positive patients in early stages.

In this sense, nano biosensors are very influential measurement devices that can make the recognition procedure of significant scientific bacteria and viruses easy, rapid, and active by detecting related parameters that can be interrelated to the infectious processes.

But the most important device for the detection of viruses and bacteria is colorimetric-based nano biosensors which detect the targeted viruses and bacteria very effectively with the color change phenomenon without wastage of time and can be seen by the naked eye and it can also be linked with AI for a systematic approach.

## Data Availability

No datasets were generated or analysed during the current study.

## Abbreviations

Au-CNT nanohybrid: Gold-carbon nanotube nanohybrid
RSV: Respiratory syncytial virus
AgNPs: Silver nanoparticles
CdSe-ZnS-QDs: Cadmium selenide-zinc sulfide quantum dots
AuNPs-CdSe/TeS-QDs: Gold nanoparticles-cadmium selenide/tellurium sulfide quantum dots
MEF: Metal-organic framework
FET: Field-effect transistor
EIS analysis: Electrochemical impedance spectroscopy analysis
LSV analysis: Linear sweep voltammetry analysis
DPV analysis: Differential pulse voltammetry analysis
SWCNTs: Single-walled carbon nanotubes
SPCEs: Screen-printed carbon electrodes
CV: Cyclic voltammetry
SMWV: Surface modified working electrode
